# Glutamine metabolism controls amphiregulin-facilitated chemoresistance to cisplatin in human chondrosarcoma

**DOI:** 10.7150/ijbs.86116

**Published:** 2023-10-09

**Authors:** Yu-Ying Wu, Yat-Yin Law, Yu-Wen Huang, Nguyen Bao Tran, Chih-Yang Lin, Chao-Yang Lai, Yuan-Li Huang, Chun-Hao Tsai, Chih-Yuan Ko, Ming-Chih Chou, Wei-Chien Huang, Fang-Ju Cheng, Yi-Chin Fong, Chih-Hsin Tang

**Affiliations:** 1 Institute of Medicine, Chung Shan Medical University, Taichung, Taiwan.; 2 Department of Orthopedics, Chung Shan Medical University Hospital, Taichung, Taiwan.; 3 Department of Orthopedics, Penghu Hospital, Ministry of Health and Welfare, Penghu, Taiwan.; 4 School of Medicine, Chung Shan Medical University, Taichung, Taiwan.; 5 Department of Pharmacology, School of Medicine, China Medical University, Taichung, Taiwan.; 6 Graduate Institute of Biomedical Sciences, China Medical University, Taichung, Taiwan.; 7 Translational Medicine Center, Shin-Kong Wu Ho-Su Memorial Hospital, Taipei, Taiwan.; 8 Department of Medical Laboratory Science and Biotechnology, College of Medical and Health Science, Asia University, Taichung, Taiwan.; 9 Department of Sports Medicine, College of Health Care, China Medical University, Taichung, Taiwan.; 10 Department of Orthopedic Surgery, China Medical University Hospital, Taichung, Taiwan.; 11 Department of Surgery, Chung Shan Medical University Hospital, Taichung, Taiwan.; 12 Center for Molecular Medicine, China Medical University Hospital, Taichung, Taiwan.; 13 Department of Orthopedic Surgery, China Medical University Beigang Hospital, Yunlin, Taiwan.; 14 Chinese Medicine Research Center, China Medical University, Taichung, Taiwan.; 15 Department of Medical Research, China Medical University Hsinchu Hospital, Hsinchu, Taiwan.

**Keywords:** chondrosarcoma, amphiregulin, SLC1A5, GLS, cisplatin

## Abstract

Chondrosarcoma is the second most common type of bone cancer. At present, the most effective clinical course of action is surgical resection. Cisplatin is the chemotherapeutic medication most widely used for the treatment of chondrosarcoma; however, its effectiveness is severely hampered by drug resistance. In the current study, we compared cisplatin-resistant chondrosarcoma SW1353 cells with their parental cells via RNA sequencing. Our analysis revealed that glutamine metabolism is highly activated in resistant cells but glucose metabolism is not. Amphiregulin (AR), a ligand of the epidermal growth factor receptor, enhances glutamine metabolism and supports cisplatin resistance in human chondrosarcoma by promoting NADPH production and inhibiting reactive oxygen species (ROS) accumulation. The MEK, ERK, and NrF2 signaling pathways were shown to regulate AR-mediated alanine-serine-cysteine transporter 2 (ASCT2; also called SLC1A5) and glutaminase (GLS) expression as well as glutamine metabolism in cisplatin-resistant chondrosarcoma. The knockdown of AR expression in cisplatin-resistant chondrosarcoma cells was shown to reduce the expression of SLC1A5 and GLS *in vivo*. These results indicate that AR and glutamine metabolism are worth pursuing as therapeutic targets in dealing with cisplatin-resistant human chondrosarcoma.

## Introduction

Chondrosarcoma is a form of cancer that develops from chondrocytes, which are cells responsible for the production of cartilage. Chondrosarcoma is the second most common malignancy in bones, classified histologically into three grades (grades I-III) based on the degree of cellularity, nuclear polymorphism, alterations in the muco-myxoid matrix, and vascularization 1-4. Due to its high incidence of metastasis and recurrence, grade III accounts for most chondrosarcoma deaths, despite the fact that this accounts for only 15% of the cases 1, 4-6. For the past few decades, the primary treatment has been surgical excision 7, due largely to the resistance of chondrosarcoma to traditional chemotherapy and radiotherapy. Thus, there is an urgent need to develop effective treatments for metastatic and unresectable tumors.

Cisplatin is the name commonly used for cis-diamminedichloroplatinum (II) 8, a cytotoxic agent that has proven highly effective in treating a variety of tumors 9. Cisplatin is among the most efficient chemotherapeutic agents for soft tissue sarcomas, including chondrosarcoma 10, 11. When used to treat chondrosarcoma, cisplatin has been shown to increase the likelihood of survival 11; however, many patients develop resistance to the medication 11, 12. Administering cisplatin in conjunction with other medications has been shown to enhance treatment effectiveness while lessening side effects 11, 13.

Glutamine is the most prevalent amino acid in blood and muscle and an important source of carbon and nitrogen. After glutamine undergoes metabolic conversion into α-ketoglutarate (α-KG), α-KG enters the tricarboxylic acid (TCA) cycle, which generates energy for cells and maintains redox homeostasis 14. Glutamine is a signaling molecule that works in concert with intracellular pathways to promote cell proliferation and advance tumor growth 15. Pleiotropic glutamine plays a key role in tumorigenesis and the proliferation of cancer cells 16. Researchers have identified many human cancer cells that have high glutamine dependence 17, 18, which can lead to severe glutamine shortages in the tumor microenvironment 19. Several members of the solute-linked carrier families (SLC) transport glutamine across the plasma membrane. One major glutamine transporter is alanine-serine-cysteine transporter 2 (ASCT2; also called SLC1A5), which has high affinity and selectivity for glutamine 20. SLC1A5 has been identified as a candidate therapeutic target for cancer 21.

Amphiregulin (AR) is an epidermal growth factor and ligand of the epidermal growth factor receptor (EGFR), which has been linked to several physiological processes, including the control of lung morphogenesis, cell motility, angiogenesis, and bone formation 22. There is a growing body of evidence indicating a link between high AR expression and tumor progression 23-29. Researchers have also demonstrated that AR plays a role in controlling cancer cell motility, metastasis, and drug resistance 30. In our previous work, we demonstrated that AR facilitates cell invasion and angiogenesis in human chondrosarcomas 31. We have also demonstrated that AR enhances cisplatin chemoresistance by upregulating the expression of the ATP-binding cassette subfamily B member 1 gene (ABCB1) in chondrosarcoma *in vitro* and *in vivo* 32. However, little is known about the means by which cell metabolism contributes to AR-mediated drug resistance. In the current study, we determined that AR promotes chemoresistance to cisplatin via glutamine metabolism. Specifically, AR enhances SLC1A5 and glutaminase (GLS)-dependent glutamine metabolism and chemoresistance via the MEK/ERK and NrF2 signaling pathways. Therefore, AR and glutamine metabolism are promising targets to deal with cisplatin-resistant chondrosarcoma in humans.

## Materials and methods

### Materials

Human recombinant AR was purchased from PeproTech (Rocky Hill, NJ, USA). The detailed source of antibodies, siRNAs, and pharmacological inhibitors are listed in Supplementary Table S1-3. AR and SLC1A5 shRNAs were purchased from the National RNAi Core Facility (RNAi Core, Academia Sinica, Taiwan).

### Cell culture

Cells from the human chondrosarcoma cell line SW1353 were obtained from the American Type Cell Culture Collection (Manassas, VA, USA), and JJ012 cells were kindly supplied by Dr. Sean P. Scully (University of Miami School of Medicine). Cisplatin-resistant human chondrosarcoma SW1353 cells (SW1353-R) were selected in accordance with a protocol that we developed in a previous study 32. Chondrosarcoma cell culture conditions were prepared in accordance with a previously described protocol 33.

### 3-(4,5-dimethylthiazol-2-yl)-2,5-diphenyltetrazolium bromide (MTT) assays

Cell viability was determined via an MTT assay in accordance with the methods outlined in our previous studies 34-36. Briefly, cells were seeded in 96-well plates at a concentration of 5,000 cells per well. After treatment, the cultures were washed using PBS before adding 0.5 mg/ml of MTT solution for incubation at 37°C for 1 h. The absorbance of formazan crystals dissolved in DMSO was measured at 570 nm.

### Colony formation assays

Cells (3 × 10^3^) were seeded into 6-well culture plates. After 24 h, fresh medium with different concentrations of glucose or glutamine were added and treated with cisplatin (0.1 μM) for 14 days. Colonies were fixed using 3.7% formaldehyde and staining them with filtered 0.5% crystal violet solution for 15 min.

### RNA sequencing and data analysis

Total RNA was isolated from SW1353 and SW-1353-R cells for RNA sequencing. RNA quality and integrity were examined using a Bioanalyzer 2100 and RNA 1000 Nano LabChip Kit (Agilent). All samples with an RNA integrity number of less than 7 were excluded from the subsequent assay. After mRNA fragmentation and generation of a complementary DNA (cDNA) library, RNA sequencing was performed using an Illumina HiSeq 4000 and mapped using the HISAT package (http://ccb.jhu.edu/software/hisat2). EdgeR software was used to estimate the differentially regulated genes of all transcripts by calculating fragments per kilobase per million (FPKM). The differentially expressed genes were determined by log2 (fold change) >1 or log2 (fold change) <-1 values, and their statistical significance (p-value <0.05) was defined by R package software. The original data has been uploaded and provided with the GSE number (GSE239911).

### Quantitative real-time PCR

qPCR assays were performed using the StepOnePlus sequence detection system in accordance with an established protocol 37-39. Total RNA was extracted from chondrosarcoma cells using a TRIzol kit (MDBio, Taipei, Taiwan), and cDNA was synthesized using an M-MLV Reverse Transcriptase kit (Invitrogen, Carlsbad, CA, USA). Total cDNA (100 ng) was mixed with sequence-specific primers (Supplementary Table S4) using a KAPA SYBR^®^ FAST qPCR Kit (Applied Biosystems, Foster City, CA, USA).

### Western blot analysis

Cell lysates were prepared using RIPA buffer containing a protease inhibitor cocktail. The concentration of protein was determined using a BCA Protein Assay Kit (Thermo Fisher Scientific Inc., Rockford, IL, USA) 40, 41. Proteins were resolved using SDS-PAGE and transferred to Immobilon^®^ polyvinylidene difluoride (PVDF) membranes. The blots were blocked at room temperature using 3% BSA for 1 h prior to incubation with primary antibodies (1:3,000) for an additional 1 h. After undergoing three washes in TBST buffer, the blots were incubated with secondary antibodies. The bands were visualized using ImageQuant™ LAS 4000 (GE Healthcare, Little Chalfont, UK) 42, 43.

### Oxygen consumption rate

The oxygen consumption rate (OCR) was determined using a Seahorse XFe24 extracellular flux analyzer (Agilent Technologies) in accordance with a protocol outlined by the manufacturer. After culturing cells (1.5 x 10^4^) in 24-well plates for 24 h, the culture medium was replenished with phenol red and bicarbonate-free DMEM. The cells were then incubated at 37°C in a non-CO_2_ incubator to equilibrate the CO_2_ level in the atmosphere. The OCR was measured under baseline conditions as well as treatment conditions involving several metabolic drugs using an XFe24 analyzer. The values were normalized to cell number and analyzed using WAVE software (Agilent Technologies).

### Glutamine levels

After culturing cells (1 × 10^4^/well) in 24-well plates, the concentration of glutamine was determined using a Glutamine Detection Assay Kit (Abcam) in accordance with the manufacturer's guidelines.

### NADPH

Cells were cultured and extracted using NADPH/NADP extraction buffer. The NADPH/NADP ratio was measured using commercial NADP+/NADPH kits (Abcam).

### Reactive oxygen species levels

After culturing cells in a 96-well plate, the levels of reactive oxygen species (ROS) were measured using a ROS assay kit (Abcam) in accordance with the manufacturer's instructions.

### Immunohistochemistry staining

Tumor cell sections were deparaffinized using xylene and rehydrated with ethanol. Immunohisto-chemistry (IHC) staining was performed using a NovoLink Polymer System (Leica Microsystems) in accordance with the manufacturer's protocol. Human SLC1A5, GLS or NrF2 antibodies were added at a dilution of 1:200 and then incubated at 4°C overnight. The sections were counterstained with hematoxylin. IHC results were scored by accounting for the percentage of positive detection and the intensity of the staining in calculations using Image J software 44, 45.

### Statistics

All data are expressed as the mean ± standard deviation (S.D.). Statistical comparisons between two samples were performed using the Student's *t*-test and one-way analysis of variance (ANOVA) with *post hoc* Bonferroni correction for the comparison of multiple groups. A *p*-value of < 0.05 was considered significant.

## Results

### Glutamine metabolism supports cisplatin resistance in human chondrosarcoma by promoting NADPH production and inhibiting ROS accumulation

**Figure 1:**
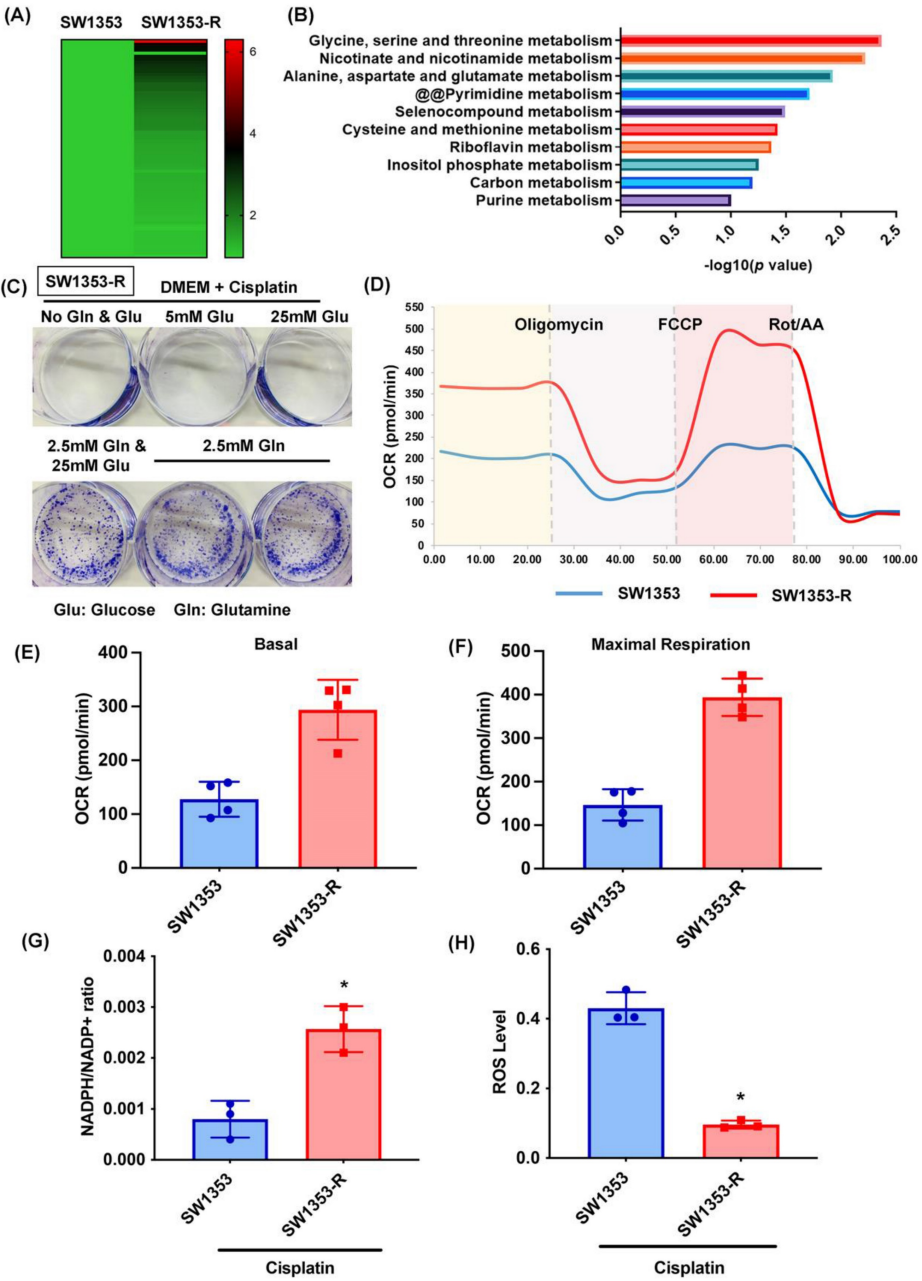
** Glutamine metabolism supports human chondrosarcoma resistance to cisplatin by upregulating NADPH production and suppressing ROS accumulation.** (A) Heat map analysis shows differentially metabolic processes in SW1353 and SW1353-R cells. (B) Functional pathways show the normalized enrichment scores of the top 10 enriched metabolism mechanisms. (C) After culturing in medium with or without glucose and glutamine, SW1353 cells were treated with cisplatin for 14 days; colony formation was then examined. (D-F) Representative trace and mean data of oxygen consumption rate (OCR) from SW1353 and SW1353-R cells. (G-H) NADPH/NADP+ ratio and ROS levels in SW1353 and SW1353-R cells. * *p* < 0.05 compared with SW1353 cells.

In a previous study, we established a new line of cisplatin-resistant human chondrosarcoma SW1353 cells that were more resistant to cisplatin treatment (SW1353-R), based on survival rates higher than those of parental SW1353 cells following cisplatin treatment 32. In the current study, we performed RNA sequencing of SW1353 and SW1353-R cells to identify the metabolic processes underlying cisplatin resistance. Heat map analysis of metabolic processes of cisplatin-resistant SW1353-R cells revealed genetic deviations from parental SW1353 cells (Fig. 1A). The metabolic mechanism associated with cisplatin resistance was identified by analyzing the top 10 metabolic mechanisms (Fig. 1B). The top two were 1) glycine, serine, and threonine metabolism and 2) nicotinate and nicotinamide metabolism, both of which involve glucose and glutamine metabolism (Fig. 1B). Thus, we sought to determine whether glucose or glutamine metabolism are involved in cisplatin resistance. In colony formation assays, cisplatin was shown to promote chondrosarcoma cell death when cultured in medium without glucose and glutamine (Fig. 1C). Culturing with glutamine but not glucose was shown to antagonize cisplatin-induced cell death (Fig. 1C). Seahorse XF technology was used to elucidate the real-time state of mitochondrial OCR. The OCR was significantly higher in cisplatin-resistant SW1353-R cells than in normal SW1353 cells (Fig. 1D-F), indicating higher energy synthesis. Tumor progression depends largely on glutamine-dependent NADPH production and ROS homeostasis 46. In the current study, the NADPH/NADP+ ratio was far higher in cisplatin-resistant SW1353-R cells than in their normal counterparts, while ROS accumulation was far lower (Fig. 1G and H). Thus, it appears that glutamine metabolism facilitates cisplatin resistance in human chondrosarcoma cells by promoting NADPH production and preventing the accumulation of ROS.

### AR promotes glutamine metabolism and supports human chondrosarcoma resistance to cisplatin

**Figure 2:**
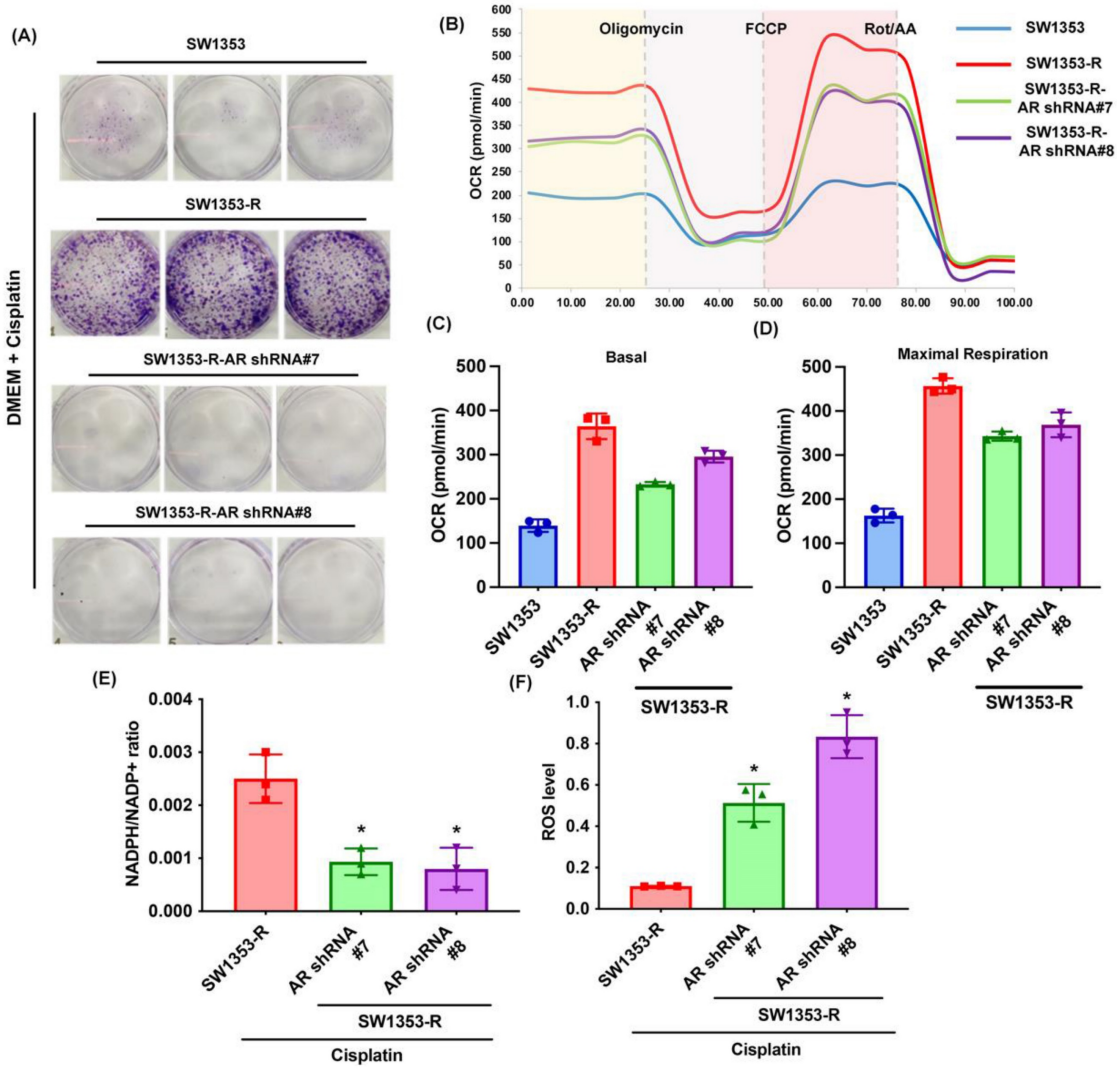
** Inhibition of AR reduces glutamine metabolism and resistance to cisplatin in chondrosarcoma.** (A) Indicated chondrosarcoma cells were treated with cisplatin for 14 days; colony formation was then examined. (B-D) Representative trace and mean data of oxygen consumption rate (OCR) from indicated chondrosarcoma cells. (E-F) NADPH/NADP+ ratio and ROS levels in indicated chondrosarcoma cells. * *p* < 0.05 compared with SW1353-R cells.

**Figure 3:**
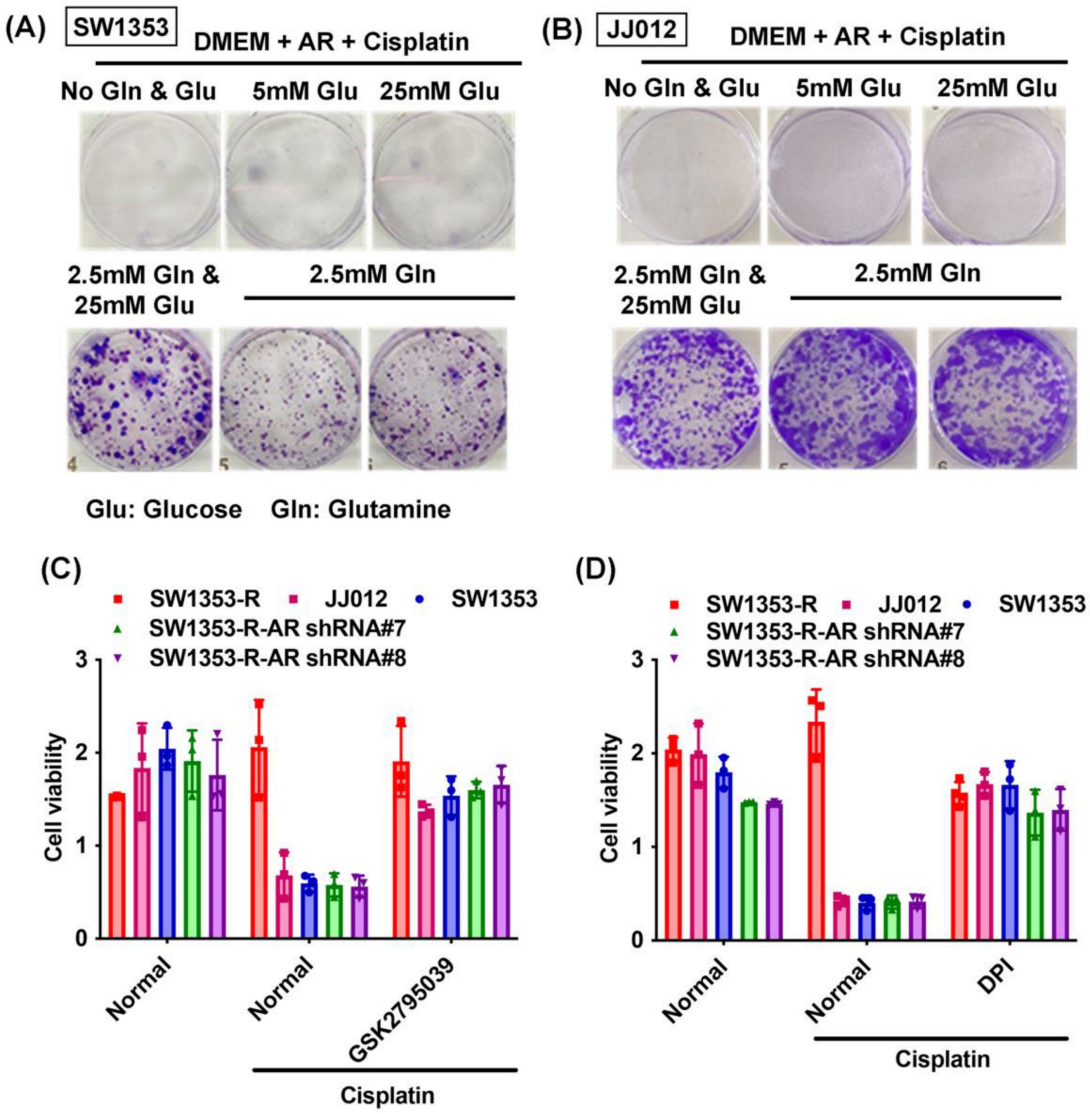
** AR enhances glutamine metabolism and resistance to cisplatin in chondrosarcoma.** (A-B) After culturing in medium with or without glucose or glutamine, chondrosarcoma cells were treated with AR and cisplatin for 14 days; colony formation was then examined. (C-D) Chondrosarcoma cells were treated with GSK2795039 or DPI and then treated with cisplatin; cell viability was examined by MTT assay.

Previous research has indicated that AR promotes cisplatin chemoresistance in human chondrosarcoma 32. In the current study, we knocked down AR expression in cisplatin-resistant chondrosarcoma cells (SW1353-R-AR shRNA cells) to determine whether AR controls glutamine metabolism. AR knockdown was shown to reverse cisplatin resistance, OCR levels, the NADPH/NADP+ ratio, and ROS accumulation (Fig. 2). Exogenous AR was shown to promote cisplatin resistance in medium containing glutamine but not glucose (Fig. 3A&B). NADPH oxidase inhibitors (GSK2795039 and diphenyleneiodonium chloride (DPI)) reversed cisplatin-induced cell death in SW1353, JJ012 and SW1353-R-AR shRNA cells (Fig. 3C&D). These results indicate that in human chondrosarcoma, AR enhances glutamine metabolism and resistance to cisplatin by increasing NADPH production and inhibiting ROS accumulation.

### SLC1A5 and GLS control AR-promoted glutamine metabolism and cisplatin chemoresistance

**Figure 4:**
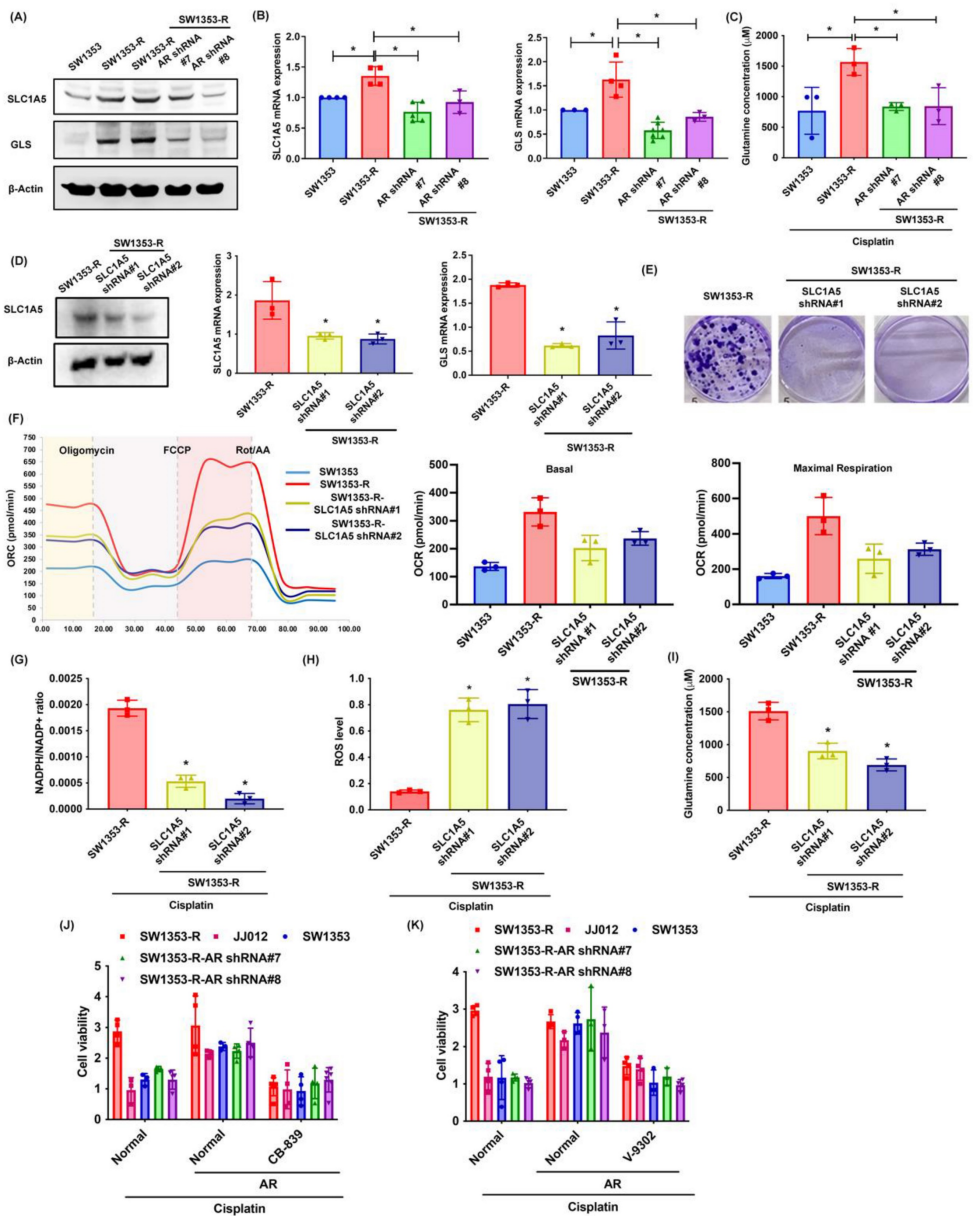
** SLC1A5 and GLS control AR-promoted glutamine metabolism and resistance to cisplatin.** (A-D) SLC1A5 and GLS expression as well as glutamine levels in indicated chondrosarcoma cells were examined. (E) Indicated chondrosarcoma cells were treated with cisplatin for 14 days, and colony formation was then examined. (F) Representative trace and mean data of oxygen consumption rate (OCR) from indicated chondrosarcoma cells. (G-I) Indicated chondrosarcoma cells were treated with cisplatin, and NADPH/NADP+ ratio, ROS levels, and glutamine concentration were then examined. (J-K) Chondrosarcoma cells were treated with CB-839 or V-9302 and then treated with cisplatin; cell viability was examined by MTT assay. * *p* < 0.05 compared with SW1353-R cells.

Glutamine is transported into cells via transporter proteins (e.g., SLC1A5) for biosynthesis into glutamate by GLS 47. The SW1353-R cells presented elevated SLC1A5 and GLS expression levels as well as elevated glutamine concentrations than SW1353 and SW1353-R-AR shRNA cells (Fig. 4A-C). Knocking down SLC1A5 expression in SW1353-R cells using SLC1A5 shRNA was shown to reduce SLC1A5 and GLS expression levels (Fig. 4D). SLC1A5 inhibition also rescued cisplatin chemoresistance, OCR levels, the NADPH/NADP+ ratio, ROS accumulation, and glutamine concentrations (Fig. 4E-I). Treatment with glutaminase inhibitor (CB-839) and glutamine transporter inhibitor (V-9302) antagonized cisplatin-induced cell death in SW1353, JJ012 and SW1353-R-AR shRNA cells (Fig. 4J&K). This means that SLC1A5 and GLS mediated the effects of AR on glutamine metabolism and resistance to cisplatin.

### MEK, ERK, and NrF2 pathways regulate SLC1A5 and GLS expression as well as glutamine metabolism

**Figure 5:**
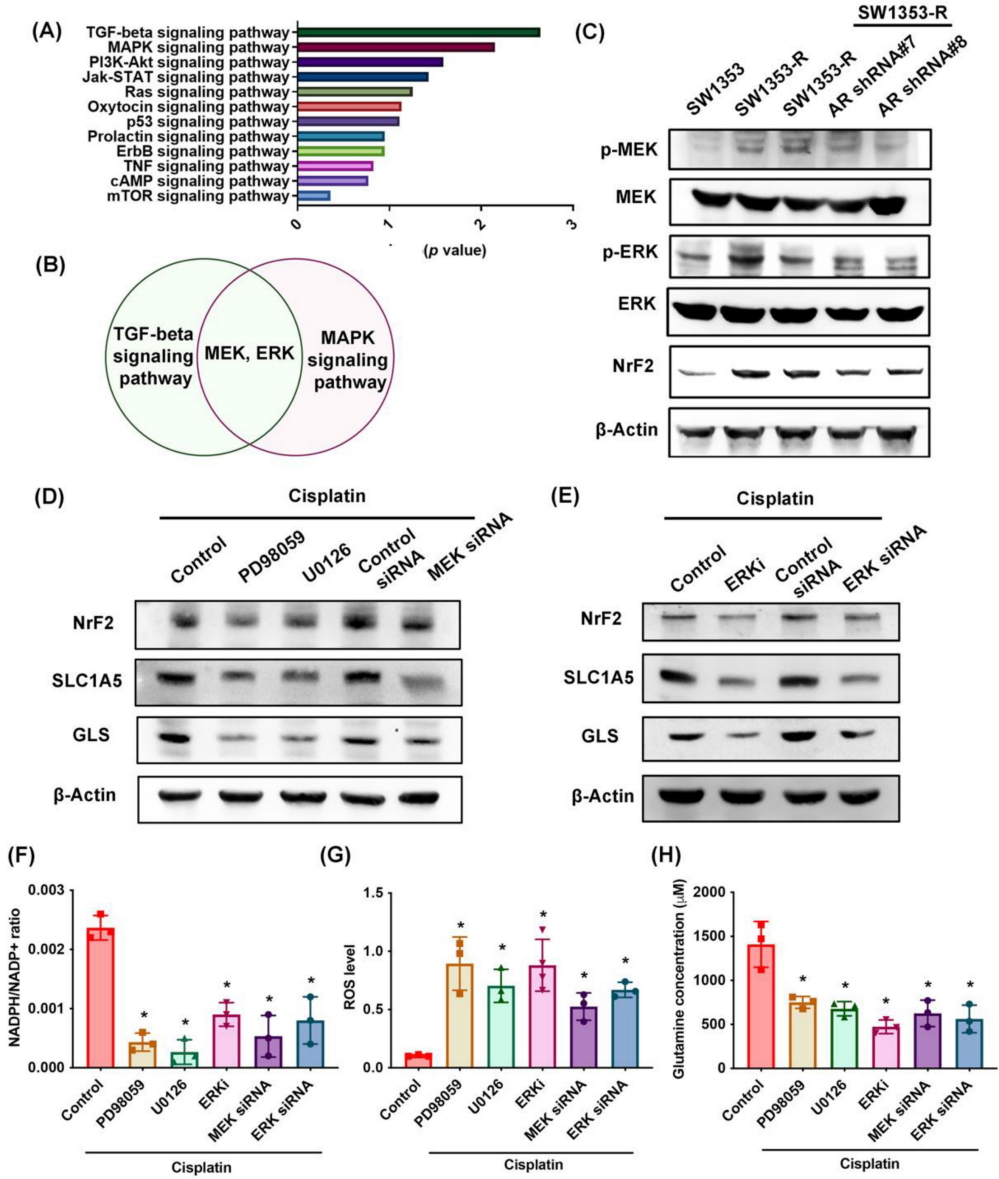
** MEK, ERK, and NrF2 pathways regulate SLC1A5 and GLS expression and glutamine metabolism.** (A) Functional pathways show the normalized enrichment scores of the top 12 enriched signaling pathways. (B) MEK and ERK pathways are involved in the top two: TGF-β and MAPK signaling pathways. (C) p-MEK, p-ERK, and NrF2 expression in indicated chondrosarcoma cells was examined. (D-H) Cells were treated with MEK and ERK inhibitors or siRNAs and then treated with cisplatin; SLC1A5 and GLS expression, NADPH/NADP+ ratio, ROS levels, and glutamine concentration were then examined. * *p* < 0.05 compared with SW1353-R cells.

RNA sequencing was used to identify potential signaling pathways involved in cisplatin resistance (Fig. 5A). We determined that the MEK and ERK pathway was associated with the top two signaling pathways, i.e., TGF-β and MAPK (Fig. 5B; Supplementary Tables S5). MEK and ERK are signaling cascades regulated the cancer chemoresistance 48. SW1353-R cells exhibited elevated p-MEK, p-ERK, and NrF2 (an antioxidant transcriptional factor) expression levels (Fig. 5C). Note that AR knockdown antagonized these effects (Fig. 5C). Treating cells with MEK or ERK inhibitors (PD98059 or U0126) or siRNAs was shown to reverse cisplatin-mediated NrF2, SLC1A5, and GLS expression, the NADPH/NADP+ ratio, ROS accumulation, and glutamine concentrations (Fig. 5D-H).

In addition, the mRNA expression of NrF2 is upregulated in cisplatin-resistant SW1353-R cells (Supplementary Fig. S1). Knockdown AR reduced the NrF2 expression (Supplementary Fig. S1). The AR regulated the mRNA expression of NrF2 in cisplatin chemoresistant can't be ruled out. These results indicate that MEK, ERK, and NrF2 signaling cascades control SLC1A5 and GLS expression as well as glutamine metabolism in cisplatin-resistant chondrosarcoma.

### AR inhibition reduced NrF2, SLC1A5, and GLS expression *in vivo*

**Figure 6:**
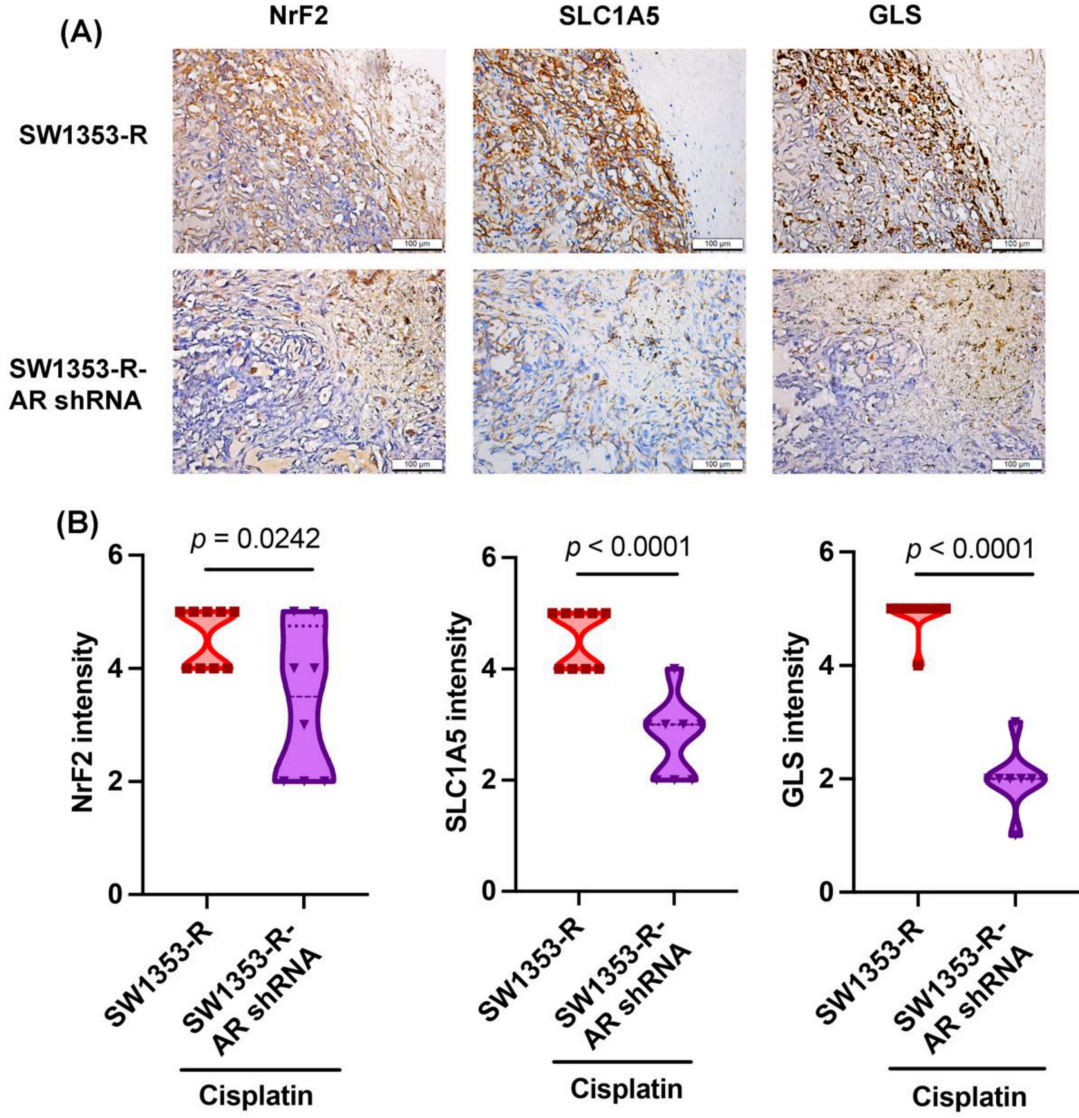
** Inhibiting AR reduces NrF2, SLC1A5, and GLS expression *in vivo*.** IHC staining detected NrF2, SLC1A5, and GLS expression.

We previously determined that AR knockdown inhibits cisplatin drug resistance *in vivo* 32. In experiments based on IHC staining, NrF2, SLC1A5, and GLS expression levels were significantly lower in the SW1353-R-AR shRNA group than in the SW1353-R group (Fig. 6), providing further evidence that AR inhibition reduces cisplatin drug resistance and glutamine metabolism *in vivo*.

## Discussion

Chondrosarcoma patients are susceptible to lung metastasis, tumor recurrence, and treatment resistance 49. The identification of reliable biomarkers and targetable molecules should make it easier to track the course of the disease and formulate a meaningful treatment plan far earlier than is currently possible 50. AR is strongly associated with oncogenesis, and in many types of cancer (e.g., breast, glioma, and lung), elevated AR expression levels are associated with a worse prognosis 51-53. In previous research, we reported that AR increases resistance to chemotherapeutic drugs, including cisplatin and doxorubicin 32, 54. In the current study on human chondrosarcoma, we determined that AR enhances glutamine metabolism and facilitates resistance to cisplatin by increasing NADPH production and inhibiting ROS accumulation. In cisplatin-resistant chondrosarcoma, the MEK, ERK, and NrF2 signaling pathways regulate AR-mediated SLC1A5 and GLS expression as well as glutamine metabolism.

A high demand for energy and raw materials alters the way that cancer cells use energy and nutrients in the biosynthesis of complex molecules for growth and proliferation (i.e., the Warburg effect) 55. Cancer cells in an oxygen-rich environment exhibit elevated glucose uptake and glycolytic activity, which is accompanied by a reduction in the contribution of glucose carbon to the TCA cycle in mitochondria 56. Some cancer cell lines (e.g., in the lung, pancreas, breast, and glioblastoma) are highly dependent on glutamine, a non-essential amino acid produced from glucose 57. In the current study, RNA sequencing identified glucose and glutamine metabolism as the two most important metabolic processes in cisplatin-resistant chondrosarcoma cells. It is interesting to note that culturing cells in medium that contained glutamine but not glucose prevented cisplatin-induced cell death. Exogenous AR was also shown to facilitate cisplatin resistance in medium containing glutamine but not glucose. Our results also demonstrated that glutamine supplement can help AR to upregulate ABCB1 expression (Supplementary Fig. S2). Therefore, we can't rule out the role of ABCB1 in AR enhanced glutamine metabolism and cisplatin resistance. To the best of our knowledge, this is the first study to provide evidence that glutamine metabolism is more important than glucose metabolism in the resistance of chondrosarcoma to cisplatin.

The ten-fold increase in glutamine uptake observed in glutamine-dependent cells suggests that the function of glutamine is not limited to that of a nitrogen donor for the production of nucleotides and amino acids 58. Other research groups have established that mitochondrial metabolism relies heavily on glutamine as a primary substrate crucial to maintaining membrane potential and integrity while facilitating the synthesis of NADPH required for redox regulation 59. Our results found that glutamine metabolism supports cisplatin resistance in human chondrosarcoma cells by promoting NADPH production and preventing the accumulation of ROS. AR was shown to facilitate glutamine-dependent cisplatin resistance in chondrosarcoma, whereas NADPH oxidase inhibitors were shown to antagonize cisplatin-induced cell death in SW1353, JJ012 and SW1353-R-AR shRNA cells. This means that AR promotes glutamine metabolism and cisplatin resistance by enhancing the production of NADPH and reducing ROS accumulation.

SLC1A5 is a glutamine transporter crucial to the functioning of cancer cells 20. Research into several forms of human cancer (e.g., breast, prostate, and lung cancer) has shown that inhibiting the expression of SLC1A5 can reduce glutamine uptake and thereby halt cell proliferation 60. In other tumor cell lines, the knockdown or pharmacological inhibition of SLC1A5 has been shown to cause cell apoptosis, making it an ideal therapeutic target 21. In the current study, we observed elevated SLC1A5 expression levels in cisplatin-resistant chondrosarcoma cells. The genetic inhibition of SLC1A5 was shown to rescue cisplatin chemoresistance, OCR levels, the NADPH/NADP+ ratio, ROS accumulation, and glutamine concentration levels. The pharmacological inhibition of SLC1A5 or GLS was shown to antagonize cisplatin-induced cell death in parental chondrosarcoma and SW1353-R-AR shRNA cells. The fact that AR inhibition reduced SLC1A5 and GLS expression *in vivo* means that AR-promoted glutamine metabolism and resistance to cisplatin are mediated by SLC1A5 and GLS. Glutamine is a precursor of glutathione (GSH), which mediates the redox homeostasis to defend cancer cells from oxidative stress 61. Here we demonstrated the roles of SLC1A5, GLS and ROS in cisplatin chemoresistance. However, we didn't examine the effects of GSH. Whether GSH also involved the cisplatin resistance in chondrosarcoma are needs further examination.

MEK and ERK are signaling pathways commonly encountered in cancer chemoresistance 48. RNA sequencing in the current study revealed that the MEK and ERK pathways play key roles in the resistance of chondrosarcoma to cisplatin. Western blotting analysis revealed that MEK and ERK phosphorylation levels were higher in cisplatin-resistant cells than in their normal counterparts. The genetic or pharmacological inhibition of MEK or ERK was shown to reverse the effects of cisplatin in terms of the NADPH/NADP+ ratio, ROS accumulation, and glutamine concentration. This means that the MEK and ERK signaling pathways regulate SLC1A5 and GLS expression as well as glutamine metabolism in cisplatin-resistant chondrosarcoma. Finally, it should be noted that limitations exist in this study. Our data strongly suggest that AR promotes glutamine metabolism and supports cisplatin resistance in human chondrosarcoma. However, we don't have tissue samples from clinic individuals, the impact of AR, SLC1A5 and GLS on cisplatin-resistant chondrosarcoma needs to be assessed in patients.

**Figure 7:**
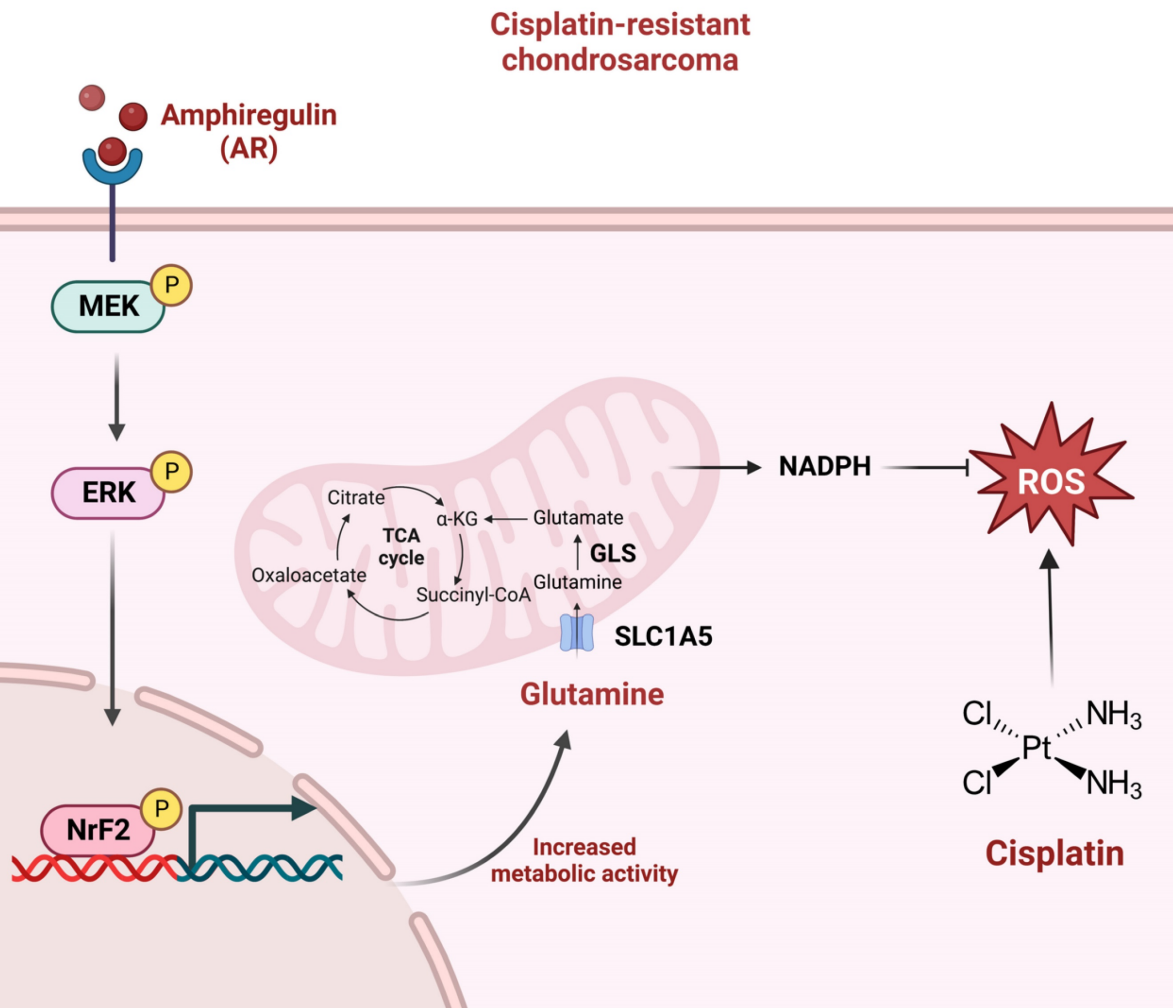
** Schematic presentation of the signaling pathways involved in AR-mediated glutamine metabolism and chemoresistance in human chondrosarcoma cells.** AR promotes glutamine metabolism and supports human chondrosarcoma resistance to cisplatin through upregulating NADPH production and diminishing ROS accumulation. MEK, ERK, and NrF2 signaling pathways control AR-mediated SLC1A5 and GLS expression as well as glutamine metabolism in cisplatin-resistant chondrosarcoma.

In conclusion, this study determined that AR promotes glutamine metabolism and supports cisplatin resistance in human chondrosarcoma by upregulating NADPH production and downregulating ROS accumulation. The MEK, ERK, and NrF2 signaling pathways were shown to control AR-mediated SLC1A5 and GLS expression as well as glutamine metabolism in cisplatin-resistant chondrosarcoma (Fig. 7). The further development of AR, SLC1A5 and GLS inhibitor or antibodies are potential candidates to treat of cisplatin-resistant chondrosarcoma. Taken together, it appears that AR and glutamine metabolism are worth pursuing as therapeutic targets for cisplatin-resistant human chondrosarcoma.

## Supplementary Material

Supplementary figures and tables.
