# Supplementary material for: Glutamine metabolism controls amphiregulin-facilitated chemoresistance to cisplatin in human chondrosarcoma
Source: Int J Biol Sci. 2023 Oct 9;19(16):5174–86. doi: 10.7150/ijbs.86116 (PMC10620823; doi:10.7150/ijbs.86116)
Supplement: Supplementary file 1 — Supplementary figures and tables. [file ijbsv19p5174s1.pdf]

Supplementary data

Supplementary Table S1. Pharmacological inhibitors were used in this study.

Target	Name	Source	Catalog No.	Concentration
**NADPH oxidase**	GSK2795039	Selleckchem	HY-18950	10 µM
**NADPH oxidase**	DPI	Selleckchem	S8639	10 µM
**Glutaminase**	CB-839	Selleckchem	S7655	1 µM
**Glutamine transpoter**	V-9302	Selleckchem	S8818	9.6 µM
**MEK**	PD98059	Sigma	P215-1MG	10 µM
**MEK**	U0126	Sigma	U120-1MG	10 µM
**ERK**	ERKi	Santa Cruz	sc-203945	10 µM

Supplementary Table S2. siRNAs were used in this study.

Gene	Species	Source	Catalog no.
**MEK**	Human	Ambion	s11168
**ERK**	Human	Santa Cruz	sc-35335

Supplementary Table S3. Antibodies were used in this study.

Protein	Dilution ratio	Source	Catalog no.
**SLC1A5**	1:2000	Cusabio	CSB-PA620892DSR1HU
**NRF2**	1:500	Santa Cruz	sc-722
**GLS**	1:2000	Sigma	HPA036223
**β-Actin**	1:3000	Sigma	A5441
**MEK**	1:500	Cell signaling	9122
**p-MEK**	1:2000	Cell signaling	2338
**ERK**	1:500	Santa Cruz	sc-1647
**p-ERK**	1:500	Santa Cruz	sc-7383

Supplementary Table S4. Primers were used in this study.

Gene	Forward	Reverse
**SLC1A5**	TCATGTGGTACGCCCCTGT	GCGGGCAAAGAGTAAACCCA
**GLS**	AGGGTCTGTTACCTAGCTTGC	ACGTTCGCAATCCTGTAGATTT
**NrF2**	TTCCCGGTCACATCGAGAG	TCCTGTTGCATACCGTCTAAATC

Supplementary Table S5. The signaling pathway in TGF-beta and MAPK

TGF-beta pathway	MAPK pathway	
ACVR1C	RAC3	NGFR
BMPR1B	PDGFRA	FGF21
FST	CACNA2D2	MAPKAPK3
INHBE	DUSP7	HSPA8
SMAD6	MAPT	TGFB2
BMP4	CSF1R	FAS
MAPK1	HSPA1A	GADD45A
NBL1	MYD88	HSPA2
BAMBI	MAP3K1	PRKACB
SMAD1	EGF	TGFA
BMP2	DUSP4	NTF3
TGFB2	CACNA1A	FGF2
ID1	MAP2K6	RASGRF1
PITX2	MAP2K2	BDNF
GDF5	MAP2K1	IL1R1
GREM1	EFNA2	RAPGEF2
RGMA	MAP3K5	HSPA1L
MAP2K2	CACNB4	FGFR3
MAP2K1	DUSP8	ANGPT4
MYC	CACNA1G	MYC
GDF6	HSPA1B	GADD45B
E2F5	PDGFB	MAPK13
BMP5	CACNA2D4	PTPN7
RGMB	RPS6KA6	CACNG8
INHBA	DDIT3	MEF2C
	MAPK1	MAPK10

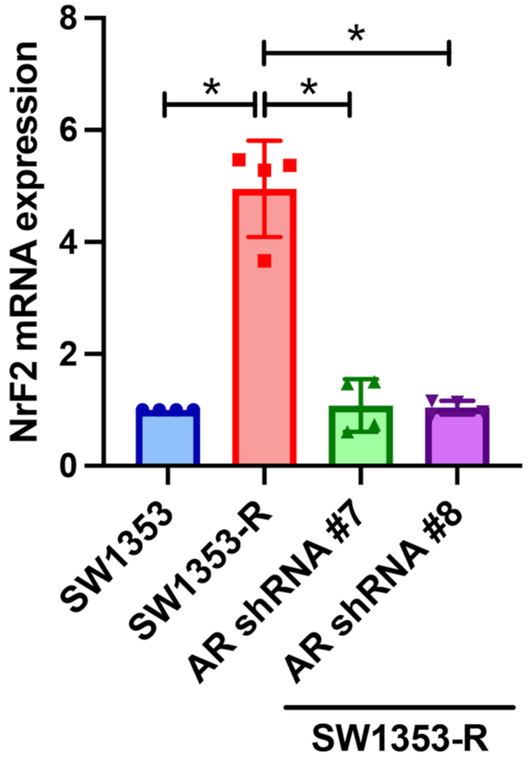

Figure S1. Knockdown AR reduces the NrF2 expression in cisplatin-resistant SW1353-R cells. The mRNA levels of NrF2 in indicated cells were examined by qPCR.

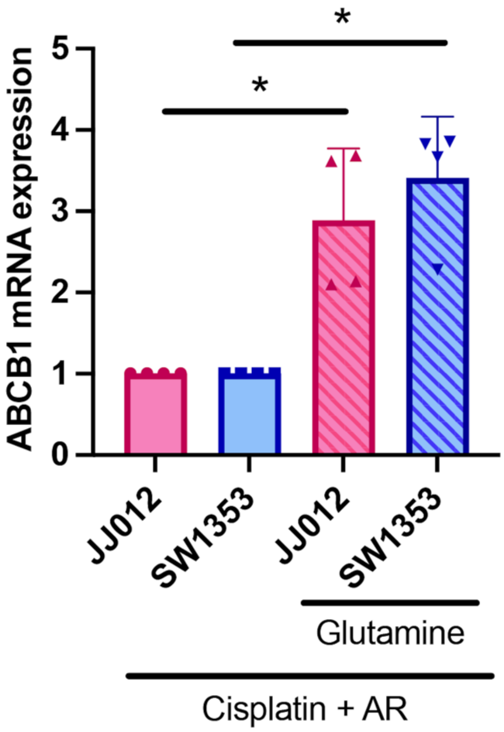

Figure S2. Glutamine supplement enhances AR to upregulate ABCB1. Cells were stimulated with AR (50 μg/ml) for 24 h, then incubated with medium containing glutamine (2.5 mM) and cisplatin (1 μM), the mRNA of ABCB1 was examined by qPCR.
